# Social, cultural and economical determinants of diabetes mellitus in Kalutara district, Sri Lanka: a cross sectional descriptive study

**DOI:** 10.1186/1475-9276-11-76

**Published:** 2012-12-13

**Authors:** Ambepitiyawaduge Pubudu De Silva, Sudirikku Hennadige Padmal De Silva, Isurujith Kongala Liyanage, Lalini Chandika Rajapakse, Kosala Saroj Amarasiri Jayasinghe, Prasad Katulanda, Chandrika Neelakanthi Wijeratne, Sumedha Wijeratne

**Affiliations:** 1 Department of Community Medicine, Faculty of Medicine, University of Colombo, Colombo, Sri Lanka; 2 Department of Para Clinical Sciences, Faculty of Medicine, General Sir John Kotelawala University, Colombo, Sri Lanka; 3 Department of Clinical Medicine, Faculty of Medicine, University of Colombo, Colombo, Sri Lanka; 4 Department of Obstetrics & Gynaecology, Faculty of Medicine, University of Colombo, Colombo, Sri Lanka

**Keywords:** Prevalence of diabetes mellitus, Social determinants of diabetes mellitus, Diabetes in plantation sector

## Abstract

**Introduction:**

Sri Lanka is a country that is expected to face a high burden of diabetes mellitus (DM). There is a paucity of data on social and demographic determinants of DM, especially in the plantation sector.

**Aims:**

To describe social and economic correlates and inequalities of DM in Kalutara District.

**Methods:**

A cross sectional descriptive study was carried out among adults over the age of 35 years. A sample of 1300 individuals was selected using stratified random cluster sampling method from 65 Grama Niladari Divisions (GND), which were representative of urban, rural and plantation sectors. Twenty households were randomly selected from each division and one adult was randomly selected from each household. Data were collected using a pre-tested questionnaire. Fasting plasma blood sugar of ≥126mg/dl was used to define DM. Significance of prevalence of diseases and risk factors across different socio-economic strata were determined by chi square test for trend.

**Results:**

Of 1234 adults who were screened (628 males), 202 (14.7%) had DM. Higher DM proportions (16.1%) were seen in the highest income quintile and in those educated up to Advanced Levels (AL) and above (17.3%). Prevalence in the urban, rural and plantation sectors were 23.6%, 15.5% and 8.5% respectively. Prevalence among Sinhalese, Tamils and Muslims were 14.4%, 29.0% and 20.0% respectively. There was a gradient in prevalence according to the unsatisfactory basic needs index of the GND with the highest proportion (20.7%) observed in the richest GND. The highest social status quintile demonstrated the highest proportion (17.4%) with diabetes mellitus.

**Conclusion:**

There is a higher prevalence of diabetes mellitus in the more affluent and educated segments of society. There is also a higher prevalence among urban compared to rural and estates. Sri Lanka is in an early stage of the epidemic where the wealthy people are at a higher risk of DM.

## Introduction

Chronic non communicable diseases are increasingly responsible for the highest burden of disease in Sri Lanka due to the demographic and epidemiological transition [1]. Type 2 DM is one of the most important chronic diseases that contributes to many adversities at individual, societal and global levels [2]. Hospital morbidity and mortality data reveal a significant increase of chronic diseases [3,4]. An exponential increase in hospitalization has been predicted for type 2 diabetes mellitus (DM), essential hypertension and ischaemic heart disease (IHD) based on hospital admission data in Sri Lanka [5].

Socio-economic status, demographic factors and ethnicity are important determinants of these diseases and adverse outcomes. Identifying these associated factors are important in controlling the disease as they provide means of identifying high risk populations and asymptomatic patients.

Sri Lanka is a multi ethnic country with a land area of approximately 65,000km^2^ where urbanization, plantations and other development projects have had a significant impact on people’s behaviours. The smallest administrative division in Sri Lanka, which is known as Grama Niladhari Divisions (village officer divisions) are categorized into three sectors; urban, rural and estate, according to the level of urbanization, infrastructure development and presence of large estates (tea or rubber). Approximately 14%, 80% and 6% of the Sri Lankans live in these sectors respectively [6]. These populations are from different socio-economic backgrounds with exposure to varied environmental factors.

Although recent community based studies are available, there is a paucity of data on social and demographic correlates of diabetes from the plantation sector. This study aimed to describe prevalence and social and economical determinants of diabetes mellitus in Kalutara District.

## Methods

This was a cross sectional study carried out in the Kalutara District, which has urban, semi-urban, rural and plantation sectors [6]. Adults who were in the age group of 35 to 64 years were selected for the study. The sample size was calculated to determine a prevalence of 16% [7] with a margin of error at 3% and α error at 5%. Since a value for roh was not available from earlier studies a cluster factor of 2 was taken. The calculated sample size of 1147 was further inflated to 1262 in order to account for a drop-out rate of 10%, which was then rounded off to 1300. Stratified multistage cluster sampling was used. Since people of similar socioeconomic status tend to cluster together, the cluster size was restricted to twenty to accommodate more clusters in the study. This was expected to produce a wider scatter of the sample.

The first level of stratification was urban, rural and estate sectors. The rural and plantation sectors were over sampled in order to ensure adequate numbers of subjects with DM from these sectors. The final sample consisted of 600 individuals from the rural sector, 400 from urban sector and 300 from estate sector.

The primary sampling unit was the Grama Niladari Division (GND) (village officer division). GNDs were randomly selected within each sector. Probability Proportionate to Size sampling method was used and the probability of a GND being selected was proportionate to its population of 35 to 64 year olds. In each GND, individual households were selected randomly using the electoral registry.

Only those who had resided in the selected household for a continuous period of more than one year were included in order to ensure that all participants had an established lifestyle, related to the area of residence. All the adults who are eligible for the study in each household were listed and one individual was selected randomly. If the selected individual was not available, subsequent visits were made at a time he/she is available for data collection. Those living in institutions, pregnant and lactating females, and subjects on prolonged treatment with drugs known to cause diabetes mellitus were excluded.

An interviewer administered structured questionnaire was used as the study instrument. The tools used for the study were validated for Sri Lanka. The questionnaire was pre-tested and further improvements were made. The data collectors were trained in standard research methods and ethical principles. Fasting plasma glucose (FPG) levels were measured using venous blood samples after an overnight fast of 10 hours. Analysis was done at the Public Health Laboratory of National Institute of Health Sciences (Kalutara), using Clini Check Plus Mini Analyser using hexokinase-glucose 6 phosphate dehydrogenase method with colorimetry. Diabetes was defined as having one or more of the following criteria

● Study participants with FPG level of ≥126 mg/dl [8]

● Currently (within the past 4 weeks) on insulin

● Currently (within the past 4 weeks) on hypoglycaemics

FPG ≥100mg/dl but <126mg/dl is considered as Impaired Fasting Glucose (IFG) [8]. Measures were taken to improve the quality and accuracy of data in the design and implementation stages of the study. Steps were taken to minimize sampling and measurement errors including quality control of the laboratory analysis of FPG. These included using a block design to assess the variation between instruments and observers and measuring the correlation coefficients of inter and intra assay precision checks for plasma glucose.

Social status index was assessed as described by De Silva [9] and Unsatisfactory Basic Needs Index (UBNI) as described by Satharasinghe in 2008 [10].

After estimating crude prevalence, weight adjustments were made to correct for different probabilities. All results presented for the district are weighted and prevalence standardised for age and sex. Significance of prevalence of diseases and risk factors across different socio-economic strata were determined by chi square test for trend.

## Results

Of 1,300 individuals who were selected for the study, 1,234 (94.92%) participated and 38 individuals could not be traced in the community despite three home visits by the data collectors. Consent was refused by 28 individuals. Seven participants changed residence during the time of data collection and were thus excluded from the final analysis as specified in the protocol. Data from 1,227 (94.39%) individuals was analysed. The age and sex distribution of the study sample closely resembles that of the study population living in study area (Table 1).

**Table 1:** Age and sex distribution of the study population in Kalutara district and the study sample

**Characteristics**	**Population**		**Study Sample**	
	**Number**	**Percentage**	**Number**	**Percentage**
**Sex**				
Male	174,539	48.6%	656	49.1%
Female	184,215	51.4%	644	50.9%
**Age**				
35 to 39	77,893	21.7%	214	20.7%
40 to 44	70,994	19.8%	231	20.0%
45 to 49	64,234	17.9%	228	17.7%
50 to 54	60,994	17.0%	209	17.1%
55 to 59	48,024	13.4%	232	13.5%
60 to 64	36,615	10.4%	186	10.9%
**Total**	**358,754**	**100.0%**	**1,300**	**100.0%**

Age and sex adjusted prevalence of diabetes mellitus and IFG was 14.7% and 14.3% respectively. Prevalence of diabetes mellitus increased with increasing age up to the 55-59 year age category and decreased thereafter. This increase in prevalence is statistically significant (p for trend <0.0001) over age categories when the last two age groups are merged (55-64 years) This trend remained significant for both sexes. (Table 2).

**Table 2:** Age and sex distribution of diabetes mellitus among adults in Kalutara

	**Diabetes status (n = 1234)**					
	**Normal (n = 832)**		**IFG (n = 200)**		**DM (n = 202)**	
	**Number**	**Percent**	**Number**	**Percent**	**Number**	**Percent**
**Male**						
35 to 39 Years	87	87.7%	15	9.8%	5	2.5%
40 to 44 Years	85	77.7%	13	11.1%	13	11.2%
45 to 49 Years	70	81.2%	18	7.2%	19	11.6%
50 to 54 Years	66	67.4%	12	7.7%	22	24.9%
55 to 59 Years	69	64.1%	24	12.8%	20	23.0%
60 to 64 Years	60	69.3%	11	14.7%	19	16.0%
**Female**						
35 to 39 Years	65	81.7%	8	11.4%	6	6.9%
40 to 44 Years	79	72.0%	15	14.6%	16	13.4%
45 to 49 Years	72	62.0%	18	17.8%	20	20.2%
50 to 54 Years	58	58.5%	26	29.0%	18	12.5%
55 to 59 Years	67	50.0%	23	26.0%	23	24.0%
60 to 64 Years	54	64.9%	17	14.2%	21	20.9%

The prevalence of IFG shows a steady increase with increasing level of education (Table 3).

**Table 3:** Socioeconomic determinants of fasting glucose levels among adults in Kalutara, Sri Lanka

**Characteristic**	**Glucose tolerance (n = 1234)**						**p**
	**Normal (n = 832)**		**IFG (n = 200)**		**DM (n = 202)**		
	**Number**	**Percent**	**Number**	**Percent**	**Number**	**Percent**	
**Sex**							
Male	437	75.7%	93	10.2%	98	14.1%	*0.236*
Female	395	66.6%	107	18.2%	104	15.2%	
**Age Category**							<0.001 *
35 to 39 Years	152	84.6%	23	10.6%	11	4.8%	
40 to 44 Years	164	74.7%	28	13.0%	29	12.3%	
45 to 49 Years	142	71.4%	36	12.6%	39	16.0%	
50 to 54 Years	124	62.9%	38	18.6%	40	18.6%	
55 to 59 Years	136	58.0%	47	18.5%	43	23.4%	
60 to 64 Years	114	66.9%	28	14.4%	40	18.7%	
**Ethnicity**							
Sinhalese	554	71.2%	130	14.4%	156	14.4%	
Tamil	208	60.6%	50	10.4%	23	29.0%	
Muslim	69	68.5%	19	11.6%	22	20.0%	*0.003*
Other	01	91.5%	01	3.3%	01	05.2%	
**Sector**							
Urban	224	58.6%	68	17.8%	90	23.6%	
Rural	397	69.9%	83	14.6%	88	15.5%	0.001*
Plantation	211	74.3%	49	17.3%	24	08.5%	
**Education Category**							
No schooling	35	73.8%	07	9.8%	06	16.4%	
Grade 5 or below	199	76.5%	53	10.9%	31	12.6%	
Grade 6 to 10	274	70.5%	56	13.4%	78	16.1%	0.019*
O/L to Grade 12	190	72.9%	49	15.3%	45	11.8%	
AL and above	91	66.9%	25	15.8%	31	17.3%	
**Occupation Category**							
Professional	07	97.5%	02	1.0%	03	1.4%	
Technical & clerical	38	72.3%	11	15.3%	12	12.4%	
Vendors & sellers	82	60.0%	23	14.3%	31	25.6%	*0.175*
Skilled manual workers	160	84.8%	24	7.9%	21	7.2%	
Unskilled manual workers	181	74.8%	36	14.3%	23	10.8%	
Retired	35	60.2%	15	19.7%	14	20.1%	
Unemployed	40	70.6%	8	6.5%	11	22.9%	
Housewife	257	65.5%	76	18.1%	82	16.4%	
**Income Category (per month)**							
<10,000	293	73.2%	66	12.4%	53	14.3%	
10,000 to 30,000	440	70.3%	107	14.5%	113	15.1%	0.005*
>30,000	74	64.7%	23	19.2%	35	16.1%	
**Social status index**							
1^st^ quintile	158	66.3%	38	16.3%	47	17.4%	
2^nd^ quintile	153	71.1%	39	14.5%	51	14.5%	
3^rd^ quintile	158	73.2%	39	12.0%	45	14.7%	0.006*
4^th^ quintile	163	71.7%	38	14.7%	42	13.6%	
5^th^ quintile	182	76.1%	43	16.9%	17	7.0%	
**UBNI**							
1	80	91.0%	07	4.7%	08	4.3%	
2	119	93.6%	34	1.4%	13	5.0%	
3	176	70.8%	44	16.9%	28	12.3%	< 0.001*
4	214	71.6%	46	13.6%	64	14.7%	
5	243	63.3%	69	16.0%	89	20.7%	

*chi square test for trend was used. IFG: Impaired Glucose Tolerance, DM: Diabetes Mellitus, DM, IFG, Impaired Fasting Glucose; UBNI, Unsatisfactory Basic Needs Index.

Prevalence of both diabetes mellitus (DM) and IFG increased with income (p for trend < 0.05). The prevalence of DM was highest among the people with the highest (1^st^ quintile) social status index (SSI). There was a statistically significant inverse relationship between the SSI and prevalence of DM. A similar trend was observed for UBNI.

Prevalence of DM was higher in females but this was not statistically significant. However females showed a statistically significantly higher prevalence of IFG (p < 0.001) when compared to males. Prevalence of both DM and IFG increased with income. However, a definitive pattern was not observed for different levels of education.

The prevalence of IFG shows a “U” shaped distribution when plotted against SSI, with high figures in the 1^st^ and 5^th^ quintiles and lowest in the 3^rd^ quintile. A clear pattern is not visible in the distribution of the prevalence of IFG by UBNI. The highest prevalence of IFG was observed in the 3^rd^ category of UBNI.

## Discussion

The response rate of study participants was 94.4%, which is considered satisfactory in community based surveys. The study population is comparable to the population living in study area which indicates that the sampling and sample weighting applied were accurate.

The study showed that the overall prevalence of DM was 14.7% for adults aged between 35 to 64 years. In early 1990’s the prevalence of DM in the rural sector was 2.5% [11] while in the suburban populations this was 5.0% [12]. Prevalence in urban areas remained as high as 13.9% during the mid 1990s [13]. Above data are from studies which were carried out in small geographical areas involving small sample sizes and the prevalence reported in these were not standardized for age and sex. A decade later a larger survey reported a prevalence of 14.2% among persons of 30 to 64 years age group [7] and 10.9% on those above 20 yeas age [14].

A nationally representative study done by Katulanda et al. showed a higher prevalence of diabetes mellitus among urban populations (16.2%) when compared to rural populations (8.7%). It should be noted that the earlier studies used previous WHO criteria which had higher cut off levels of blood glucose for the diagnosis of diabetes. However, Katulanda et al. (2006) described the temporal trends as an increase in the diabetes prevalence both rural and urban sectors. Therefore the increase in prevalence of diabetes mellitus cannot be totally attributed to the change of diagnostic criteria alone.

Females had a higher prevalence of DM compared to males but there was no statistically significant difference between the two genders (14.1% against 15.2%) while for IFG it was statistically significant 10.2% against 18.2%. In contrast, previous studies showed a male preponderance of both DM and IFG [7,13].

The study also showed a higher prevalence of DM among Tamils. This was mainly among Tamils living in the urban sector. However the prevalence was lower in the plantation sector. Residents of plantation sector, although belong to Tamil ethnicity lead a different lifestyle. However, further studies are needed to explain this trend. Other studies have reported a higher prevalence among Muslims [15].

The highest prevalence of diabetes mellitus and IFG was observed in the urban sector (Table 2). This is likely to be due to the high prevalence of risk factors in this sector. A higher prevalence rate of diabetes in urban dwellers compared to rural persons has been a consistent finding throughout the past two decades [11,13,14].

The observed increase in the prevalence of diabetes mellitus with income corresponds with findings of previous studies. A similar finding was observed with the pre diabetic state (IFG). Illangasekera in a study carried out at Hindagala, Kandy demonstrated higher prevalence of diabetes mellitus among people with a higher socioeconomic background where the highest prevalence was observed among people in the highest quintile of the social status [15]. In this study, the higher rates of DM in the urban sector was attributed to the higher socioeconomic level of the study population and the presence of risk factors [11]. Similarly, the area-based analysis of this study found that the more affluent areas have a higher prevalence of DM in comparison to deprived areas. In contrast, data from Europe, Australia and Northern America, consistently shows prevalence rates to be higher in the lower social classes and in more deprived areas [16].

## Conclusion

This paper demonstrates the absence of a definitive pattern in the prevalence of diabetes, and impaired glucose tolerance for majority of the indices used to measure socioeconomic status. The prevalence of IFG was higher among females and a Tamil population living in urban areas had a higher prevalence. The highest prevalence of diabetes mellitus and IFG was observed in the urban sector, among people with the highest income and in the rich GN divisions.

## Competing interests

None of the authors of this paper have financial or non financial competing interests with regards to the content of this paper.

## Authors’ contributions

APDS gave the original idea, developed the methodology, conducted the training and validation of data collection, data analysis and writing of the manuscript. SHPDS entering developed the methodology, supervised data collection, conducted analysis and writing the manuscript. IKL developed the methodology, supervised data collection, conducted analysis and writing the manuscript. KSAJ also gave the original idea, developed the methodology, data analysis and writing of the manuscript. LCR also gave the original idea, developed the methodology, data analysis and writing of the manuscript. PK developed the methodology, data analysis and writing of the manuscript. CNW developed the methodology, data analysis and writing of the manuscript. SW developed the methodology, data analysis and writing of the manuscript. All authors read and approved the final manuscript.
